# Arterial hypertension and type 2 diabetes are frequent in iNPH

**DOI:** 10.1186/2045-8118-12-S1-P40

**Published:** 2015-09-18

**Authors:** Okko T Pyykkö, Ossi Nerg, Anne M Koivisto, Tuomas Rauramaa, Mikko Hiltunen, Juha E Jääskeläinen, Ville Leinonen

**Affiliations:** 1 Neurosurgery of NeuroCenter, Kuopio University Hospital, Kuopio, Finland; 2 Neurology of NeuroCenter, Kuopio University Hospital, Kuopio, Finland; 3 Department of Pathology, Kuopio University Hospital, Kuopio, Finland; 4 Unit of Clinical Pathology and Forensic Medicine, Institute of Clinical Medicine, University of Eastern Finland, Kuopio, Finland; 5 Unit of Neurology, Institute of Clinical Medicine, University of Eastern Finland, Kuopio, Finland

## Introduction

Association of hypertension and diabetes with idiopathic normal pressure hydrocephalus (iNPH) has been described earlier, however, the prognostic role of these comorbidities is unknown.

## Methods

All available hospital records and causes of death were reviewed retrospectively from a cohort of 283 iNPH patients with a median follow-up of 5.6 years (range 0.04–19.9 years) from a defined population in Middle and Eastern Finland.

## Results

A total of 148 patients (52.3%) were hypertensive and 65 patients (23.0%) diabetic. Both diseases were equally distributed among sexes, and no age differences were observed between groups. In addition, short-term shunt-response was similar with a total of 85.1% of the 269 shunted patients showing a positive response. Mortality between diabetic and non-diabetic patients was similar (58.5% vs. 48.6%, p = 0.16), however, median survival time was significantly lower in patients with diabetes compared to non-diabetic iNPH patients (6.3 vs. 9.8 years, log rank test p < 0.001). No statistically significant differences of mortality or survival time were observed in hypertensive vs. non-hypertensive patients. The most frequent cause of death among all groups was coronary heart disease.

## Conclusions

Both hypertension and diabetes are common in iNPH. A comorbid type 2 diabetes is a significant risk factor for earlier death.

